# Comparison of two acidophilic sulfidogenic consortia for the treatment of acidic mine water

**DOI:** 10.3389/fbioe.2022.1048412

**Published:** 2022-11-29

**Authors:** Tayná Diniz Frederico, Ivan Nancucheo, Werica Colaço Barros Santos, Renato Renison Moreira Oliveira, Daniella Cardoso Buzzi, Eder Soares Pires, Patricia Magalhães Pereira Silva, Adriano Reis Lucheta, Joner Oliveira Alves, Guilherme Corrêa de Oliveira, José Augusto Pires Bitencourt

**Affiliations:** ^1^ Instituto Tecnológico Vale, Belém, Brazil; ^2^ Facultad de Ingeniería, Arquitectura y Diseño. Universidad San Sebastián, Concepción, Chile; ^3^ REDEMAT/Universidade Federal de Ouro Preto (UFOP), Ouro Preto, Brazil; ^4^ Instituto SENAI de Inovação em Tecnologias Minerais, Belém, Brazil

**Keywords:** acidophilic sulfate-reducing bacteria, bioreactors, acid mine drainage, biological treatments, sulfate treatment

## Abstract

Sulfate-reducing bioreactors are a biotechnological alternative for the treatment of acid mine drainage (AMD). In this study, two separate bioreactors with pH and temperature-controlled (Bio I and II) were operated with two different acidophilic microbial consortia to determine their efficiencies in sulfate removal from a synthetic acidic mine water. The bioreactors were operated for 302 days in continuous flow mode under the same parameters: fed with a sulfate solution of ∼30 mM with a pH of 2.5, the temperature at 30°C, stirred gently at 40 rpm and using a continuous stream of nitrogen to help remove the H_2_S produced in the bioreactor. The glycerol consumption, acetate production, and sulfate removal were monitored throughout the course of the experiment. The community composition and potential metabolic functional groups were analyzed *via* 16S rRNA partial gene sequencing. Bio I consortium reduced the sulfate, achieving a range of sulfate concentration from 4.7 to 19 mM in the effluent liquor. The removal of sulfate in Bio II was between 5.6 and 18 mM. Both bioreactors’ communities showed the presence of the genus *De* *sulfosporosinus* as the main sulfate-reducing bacteria (SRB). Despite differences in microbial composition, both bioreactors have similar potential metabolism, with a higher percentage of microorganisms that can use sulfate in respiration. Overall, both bioreactors showed similar performance in treating acidic mine water containing mostly sulfate using two different acidophilic sulfidogenic consortia obtained from different global locations.

## Introduction

Acid mine drainage (AMD) is one of the major environmental problems caused by the mining industry and can cause local and regional impacts if not properly treated. AMD is wastewater that generally contains an elevated concentration of dissolved metals and metalloids, high sulfate concentration, and a low pH (Nordstrom and Alpers, 1999; Younger et al., 2002; Johnson and Halberg 2005; González et al., 2019). Their inappropriate treatment could lead to severe damage to the health of many organisms and the environment (Simate and Ndlovu, 2014). In order to prevent the damages of AMD and enhance ecological sustainability, a proper treatment and management system is required.

Conventional treatment to treat AMD involves the addition of lime, allowing the increase of pH and leading to a variety of metal ions to precipitate (Johnson and Hallberg, 2005). Despite increasing the effluent pH, this method is disadvantageous due to their high operational costs of lime transport and application. Besides, the metal-rich sludge generated must be removed from landfill areas, and the removal of sulfate sometimes is not enough to meet environmental regulations to permit the discharge of processed water into streams and rivers (Coulton et al., 2003; Johnson and Hallberg, 2005; Nancucheo and Johnson, 2014; Hernández et al., 2022). Moreover, this AMD chemical treatment does not allow the recovery of soluble metals.

In the pursuit of a more sustainable-regarded approach, the use of acidophilic sulfate-reducing bioreactors for AMD treatment has been developed at laboratory scale (Johnson and Sánchez-Andrea, 2019). In this process, sulfate-reducing bacteria (SRB) can be used to promote the recovery of valuable industrial metals, such as copper, and also to remove sulfate. Under acidic conditions, this process is a proton-consuming reaction, enabling to increase the pH of the effluent (Nancucheo et al., 2017). Furthermore, dissimilatory sulfate reduction releases hydrogen sulfide that reacts with dissolved metals, promoting their immobilization *via* precipitation of insoluble metal sulfides, such as copper sulfides (Colipai et al., 2018).

Most known SRB species are neutrophilic and have a narrow optimum growth pH (between 6 and 8), and therefore these bacteria need to be protected from direct exposure to AMD (Johnson and Santos, 2020). In addition, previous studies have shown that some SRB are acidophiles (Alazard et al., 2010; Sánchez-Andrea et al., 2015), though further research is necessary to evaluate the performance of the treatment of AMD.

This study aims to compare the performance of two acidophilic microbial consortia in bioreactors fed with a synthetic acid mine drainage, containing mostly sulfate and based on the chemical composition of wastewater from a Brazilian acidic mine water. The glycerol consumption, acetate, and sulfate concentration were measured throughout the 302 days of the experiment as well as the community composition and metabolic functional groups of the two microbial consortia were assessed.

## Materials and methods

### Set up and operation of the sulfidogenic upflow biofilm bioreactors

Two microbial consortia from different locations were used. A novel consortium (Bioreactor I; Bio I) obtained from acidic sediment of a river impacted by an abandoned sulfur mine in the Chilean Altiplano (Guerra et al., 2016; González et al., 2019) was used. A second consortium used (Bioreactor II; Bio II), obtained from mine sites in Spain and Wales (Rowe et al., 2007; Santos and Johnson, 2018), was kindly provided by Dr. Barrie Johnson, from Bangor University, United Kingdom. The set-up for the two bioreactors was the same.

Two continuous flow bench-scale bioreactors populated with each consortium were set up based on the system described previously (Santos and Johnson, 2017). A FerMac 310/60 (Eletrolab, UK) monitored the conditions from each sulfidogenic bioreactor. Each reactor was maintained at 30°C and operated at 40 rpm stirring speed. A continuous sparging (150 mL min^-1^) of sterile (0.22 μm filter) oxygen-free nitrogen gas was used to provide the anoxic conditions and remove the H_2_S produced by the SRB.

Each consortium was immobilized on porous sterile glass beads (1–2 mm diameter) that occupied ∼50% of the bioreactor vessels working volumes. The beads were covered with 800 ml of acidophilic SRB medium containing as described (Nancucheo et al., 2016): 1) autotrophic basal salts and trace elements; 2) glycerol (3 mM); 3) 0.01% (w/v) yeast extract; 4) zinc sulfate (0.02 mM); 5) 4 mM of magnesium sulfate. The inoculated medium was maintained in batch mode for 40 days for both sulfidogenic systems recirculating the beads within the bioreactor to encourage attachment and colonization of the beads. After the start-up period of each bioreactor, the continuous operation was initiated with a flow rate of 70 ml.h^−1^. The bioreactors were fed with synthetic AMD (sAMD) (Santos and Johnson, 2017), with pH adjusted to 2.5 with 5 mM glycerol as carbon and electron donor and 0.01% (w/v) yeast extract. The experiment was carried out for 302 days under continuous flow mode. The sulfate concentration in the feed liquor was maintained at ∼30 mM throughout the experiment. Samples from the effluent were taken periodically and filtered with a 0.22 μm sterile filter (Merck Millipore, United States) and stored at 4°C prior to further analysis.

### Physicochemical analysis and bacterial cell counting

Glycerol, acetate, and sulfate concentrations from the effluent liquor were periodically determined by using ion chromatography (Dionex™ ICS-5000, Thermo Fisher Scientific, MA, United States). Glycerol concentration was quantified by using a CarboPac MA1 column coupled to an ED amperometric detector. Sulfate and acetate concentrations were measured using an IonPac AS-11 column equipped with a conductivity detector. Bacteria in the effluent were enumerated using a Neubauer chamber (Brand, Germany) and viewed with a Leica DM 3000 LED optical microscope (Leica Microsystems, Germany).

### Microbial community diversity in the bioreactors and metabolic function prediction

Diversity analysis of the microbial community in the bioreactors was performed by high throughput sequencing of the 16S rRNA gene V4 region, followed by bioinformatics analysis using QIIME2 (Bolyen et al., 2019). The samples used in this study were collected every ∼50 days and samples of each inoculum were also sequenced (each bioreactor had different priming phase; therefore, samples were taken at non-identical times**).** A total of seven samples were sequenced and analyzed for each bioreactor (Table 1).

**TABLE 1:** Sampling days for microbial diversity studies.

Time	Days of the experiment of bio I	Days of the experiment of bio II
T0	Inoculum	Inoculum
T1	56 days	87 days
T2	105 days	136 days
T3	156 days	187 days
T4	210 days	241 days
T5	252 days	283 days
T6	303 days	334 days

For genomic DNA extraction, 40 ml from liquid samples was filtered through a 0.22 μm sterile cellulose acetate filter membrane (Merck Millipore, United States). Bacteria attached to the membranes were subjected to DNA extraction using the UltraClean Microbial DNA Isolation Kit (MoBio, CA, United States), according to the manufacturer’s instructions. DNA integrity and quality were checked by gel electrophoresis. DNA was quantified by fluorescence using the Qubit^®^ 2.0 fluorometer with the QubitTM dsDNA HS Assay Kit (Invitrogen ™), following the manufacturer´s instructions.

The primers 515F (5′-GTGCCAGCMGCCGCGGTAA-3′) and 806R (5′-GGACTACHVGGGTWTCTAAT-3′) were used for the amplification of bacterial consortium 16S rRNA gene V4 region (Caporaso et al., 2012; Pylro et al., 2014). Amplicons were sequenced using an Ion Torrent™ PGMTM Hi-Q™ (Thermo Fisher Scientific, MA, United States) at the Genomic Core of the Instituto Tecnológico Vale, Belém, Brazil; following the manufacturer’s instructions for sequencing single-end libraries. After sequencing, all samples were analyzed using quantitative insights into microbial ecology QIIME2 (Bolyen et al., 2019) for Linux. Genome sequences were deposited in the NCBI database, with SRA run accessions SRR21279213 (BIO I) and SRR21279225 (Bio II) in BioProjects PRJNA872571 (BIO I) and PRJNA873019 (Bio II).

FAPROTAX version 1.2.4 (Louca et al., 2016) was used to map the studied bacterial consortia’s main predicted putative metabolic functions. FAPROTAX uses the current literature on cultured strains and converts the microbial sample’s taxonomic profile into putative functional profiles based on taxa. The results were plotted on R version 4.1.1 and RSTUDIO version 1.2.5001 (R Development Core Team, 2019; RStudio Team, 2021) with ggplot 2 package (Wickham et al., 2016).

### Statistical analysis

Alfa diversity, Beta diversity, and Principal Component Analysis (PCA) were performed by program R version 4.1.1 and RSTUDIO version 1.2.5001 (R Development Core Team, 2019; RStudio Team, 2021). Alfa diversity was performed with the package Phyloseq, ggplot2, and ape (Paradis et al., 2004; McMurdie and Holmes, 2013; Wickham et al., 2016; Zucchini et al., 2018). Beta diversity was estimated by Weighted UniFrac distance with Phyloseq (McMurdie and Holmes, 2013).

Principal Component Analysis (PCA) was performed with package devtools (Zucchini et al., 2018) and factoextra (Kassambara and Mundt, 2017) and Pearson’s correlation with package Corrplot (Wei and Simko, 2021) using the data obtained from the QIIME2 and the physical-chemical analyzes referring to the days of the samples collected.

## Results

### Operation of a low sulfidogenic bioreactor

Bacterial numbers taken from the effluent in both bioreactors varied between 1.0 × 10^4^ and 4.4 × 10^6^ mL^−1^ (Supplementary Figures S1,S2), demonstrating that the bacteria present in both consortia were able to tolerate the acidic solution used as influent sAMD. Cell numbers are planktonic bacteria which derive from the detachment of the biofilm bed (50% of the total volume of the vessel) and the variability can be explained from a random process produced by loss of large pieces of biofilm during the operation.

**FIGURE 1:**
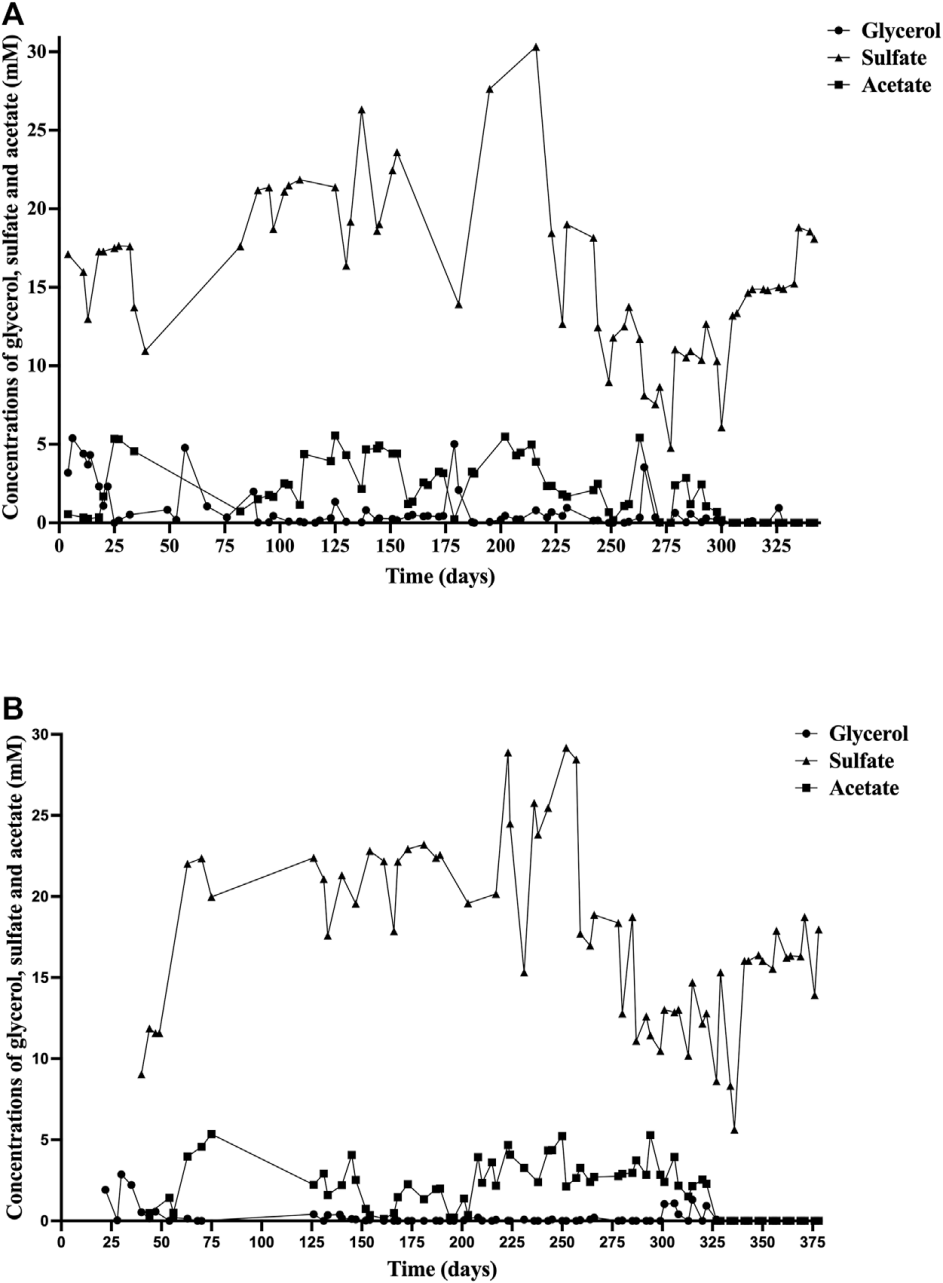
Concentrations of glycerol (○), sulfate (▲), and acetate (■) during the experiment in: **(A)** Bioreactor I (Bio I); **(B)** Bioreactor II (Bio II).

Glycerol concentrations were below 1 mM in most of the samples (Figure 1), indicating the effective consumption by the SRB as carbon source and electron donor. Acetate concentration was also quantified in the bioreactors, as the main by-product metabolite, maintaining below 5.5 mM in both bioreactors (Figure 1).

Sulfate concentration in Bio I (Figure 1A) ranged from 10.9 to 30 mM until day 216. From that day, the concentration decreased, reaching a maximum of 19 mM on day 230 and a minimum of 4.7 mM on day 277. The sulfate concentration in Bio II (Figure 1B) was maintained in a range of 9.03–29.1 mM until day 257. After that day, the concentration reached a maximum of 18.7 mM on day 258 and a minimum of 5.6 mM on day 336.

### Microbial communities’ diversity in the bioreactors

**FIGURE 2:**
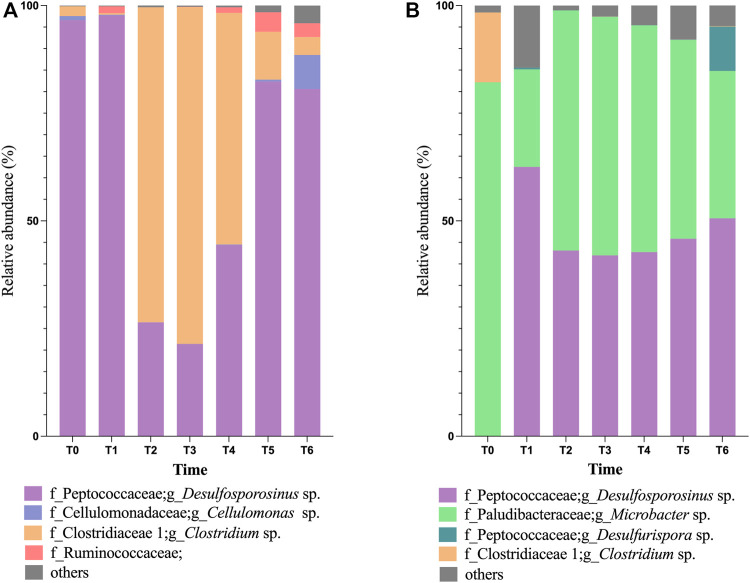
Taxonomical classification, at the genus level, of bacterial population in Bioreactor I **(A)** and Bioreactor II **(B)**, being T0 representing the inoculum population.

The most represented bacterial phylum in Bio I community (Figure 2A) was Firmicutes (>90%), followed by Actinobacteria in the inoculum (T0) and T1 (Figure 2A). The most abundant genus belonging to the phylum Firmicutes was *Desulfosporosinus*, a well-known anaerobic acidophilic SRB. Their population shifted between T2 and T4, being the most abundant again at T5 to T6. Between T2 and T4, the dominant microorganism was the Firmicutes genus *Clostridium* (Figure 2A). Microbial diversity showed low diversity in T1-T3, enhancing in T4-T6 (Supplementary Figure S3A).

The microbial community in Bio II inoculum (T0, in Figure 2B) is dominated by the phylum Bacteroidetes, with most bacteria belonging to the genus *Microbacter* (79%), followed by the genus *Clostridium* (19%). Shifts in the microbial community were observed at T1, increasing the phylum Firmicutes (66%) in detriment of Bacteroidetes (18%). Between T2-T4 sampling dates, the genus *Microbacter* was again the dominant, ranging between 51% and 62%, followed by *Desulfosporosinus* (range of 34.7%–37%). The presence of bacteria belonging to the genus *Desulfurispora* was observed at T1 and T6 sampling dates. The microbial diversity on Bio II showed variation in T1-T3, enhanced at the end of the experiment (T4 to T6) (Supplementary Figure S3B).

**FIGURE 3:**
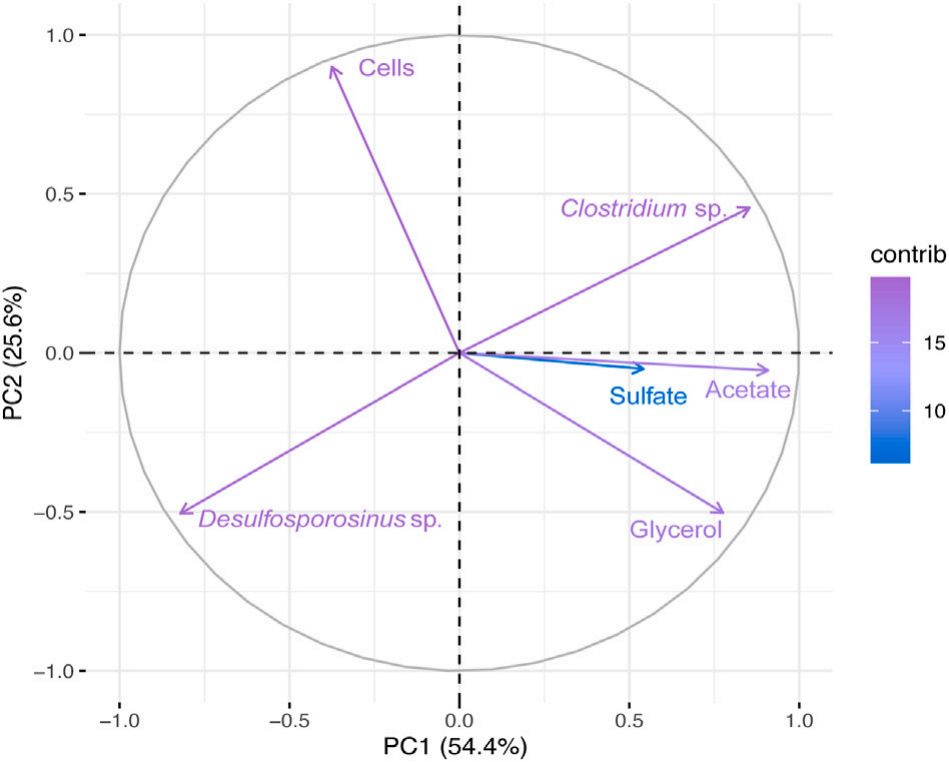
Principal Component Analyses (PCA) of the total bacterial community based on 16S rRNA gene sequences and physical-chemical analysis data from Bio I. First principal coordinates (PC1 54.4%) and the second principal coordinates (PC2 25.6%).

The PCA first principal coordinate axe (PC1) responded to 54.4% of the variability in bioreactor 1 metadata, whereas the second principal coordinates (PC2) responded to 25.6% (Figure 3). *Desulfosporosinus* and *Clostridium* showed a negative association at PCA (Figure 3) and at correlation analysis (Supplementary Figure S4A). Also, a negative correlation between microbial cells (Cells) and glycerol was observed.

**FIGURE 4:**
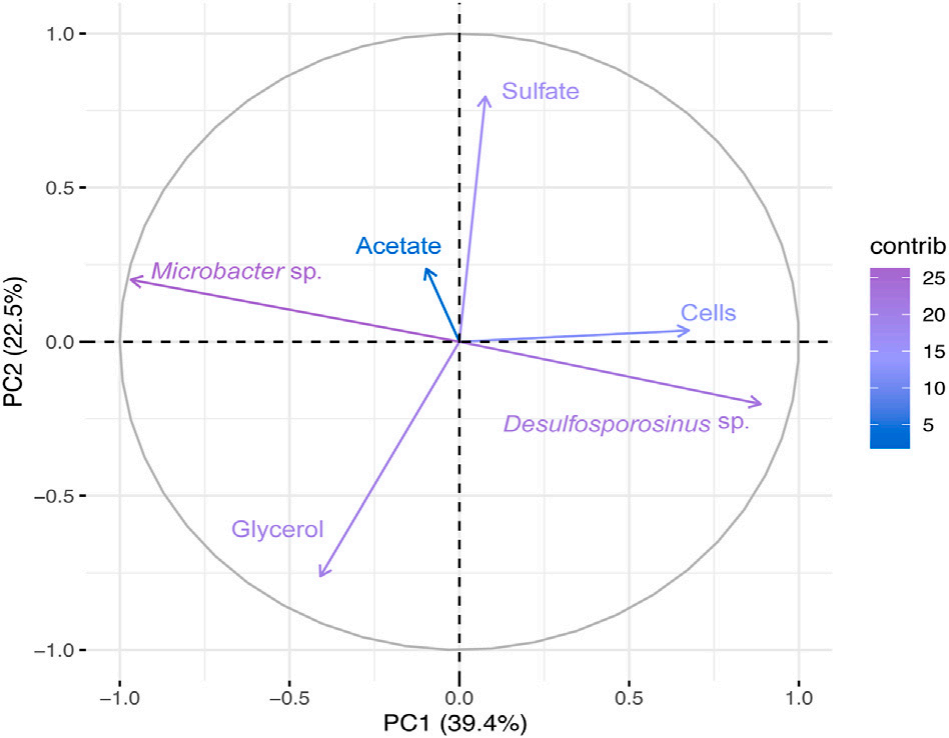
Principal Component Analyses (PCA) of the total bacterial community based on 16S rRNA gene sequences and physical-chemical analysis data from Bio II. First principal coordinates (PC1 39.4%) and the second principal coordinates (PC2 22.5%).

In Bio II (Figure 4 and Supplementary Figure S4B), the first principal coordinate axe (PC1) responded to 39.4% of the variability, whereas the second principal coordinates (PC2) responded to 22.5%. PCA shows a negative correlation between *Desulfosporosinus* and *Microbacter,* and a positive correlation between *Desulfosporosinus* and cells. The genus *Desulfosporosinus* showed a positive correlation with acetate and pH.

**FIGURE 5:**
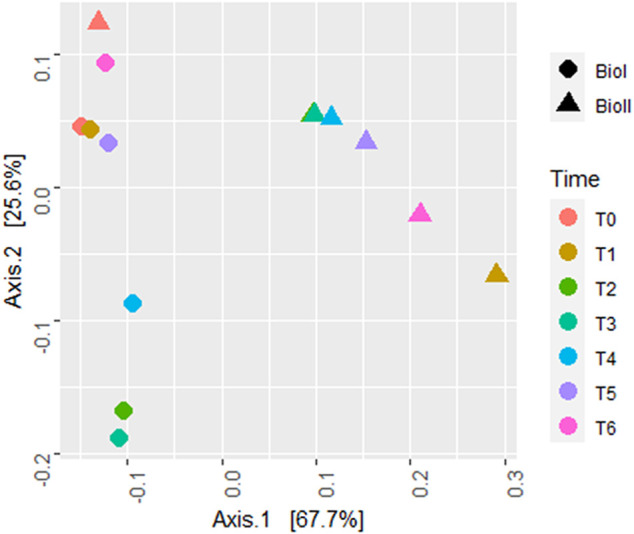
Beta diversity plot estimated by UniFrac with weights measure. Bio I (●); Bio II (▲); Each color represents a different sample time.

Beta diversity obtained by the weighted (quantitative) UniFrac distance (Figure 5) shows two clusters separating the samples by bioreactor. Only the inoculum sample (T0) from Bio II was grouped with the Bio I cluster.

**FIGURE 6:**
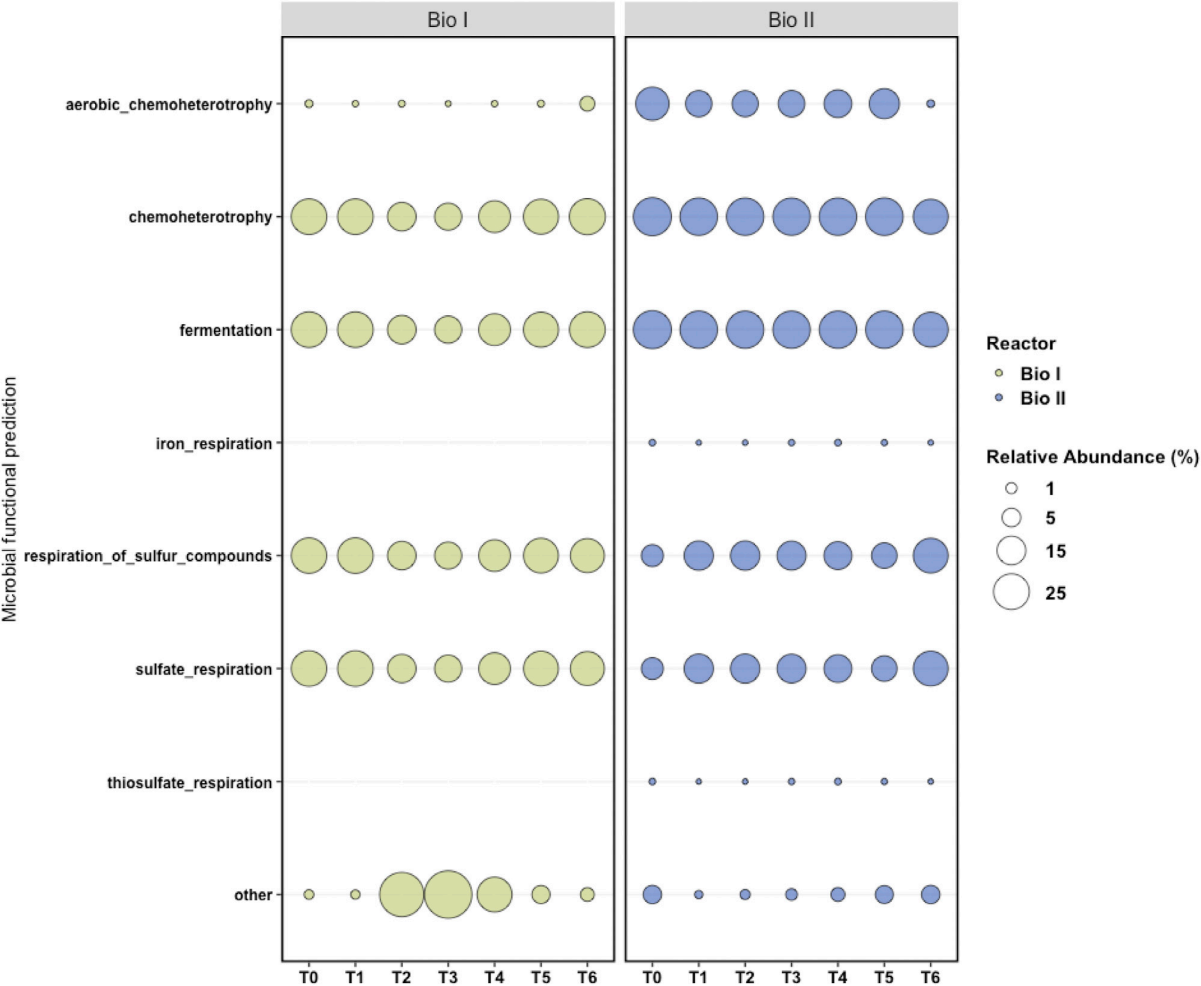
Metabolic maps generated by FAPROTAX. FAPROTAX converted taxonomic data at the level of genus and species generated by QIIME2 into functional profiles. Each column represents a sample (T0 to T6) and each line a metabolic function. In the graph, other represents the proportion of microorganisms that did not have the identified physiological functions.

The mapping of the predictive metabolic functions of Bio I (Figure 6) revealed a prevalence of microorganisms that produce cellular energy using sulfur compounds and sulfate. Furthermore, fermenters, chemoheterotrophs, a small portion of aerobic chemoheterotrophs microorganisms, and a reduction in the identification of predictive metabolic pathways were observed in samples T2, T3, and T4.

As well as Bio I, a predominance of microorganisms that use sulfur compounds to produce cellular energy, fermenters, and chemoheterotrophs was observed on the map of Bio II (Figure 6). Chemoheterotrophic microorganisms were also represented and other functions were observed in a small proportion, including iron respiration and thiosulfate respiration.

## Discussion

The mining industry frequently generates AMD, wastewater characterized by a low pH, a high concentration of dissolved metals, metalloids and sulfate (Sen and Johnson, 1999a; Hallberg, 2010; González et al., 2019). One of the main goals of this study was to compare the ability of two different SRB consortia to operate for a long term for the removal of sulfate using an acidic mine water solution.

During the operation, both bioreactors exhibited an acid-tolerant consortium with the ability to reduce sulfate under acidic conditions. Glycerol was chosen as a carbon source for being an adequate substrate for cultivating and isolating acidophilic SRB (Sen and Johnson, 1999b; Nancucheo et al., 2016; Santos and Johnson, 2017). Both consortia, in Bio I and Bio II, were efficient in consuming glycerol, maintaining below 1 mM throughout the experiment. Similar experiments carried out by Kimura et al. (2006) showed the efficiency in glycerol oxidation at pH ranging from 4.2–3.8 using an acidophilic bacteria belonging to the genus *Desulfosporosinus*.

The production of acetate concentration in Bio I and II occurs due to the presence of acetogenic SRB as *Desulfosporosinus*. One of the problems of acetogenic SRB growing at low pH is the production of acetic acid, being toxic to SRB in concentrations higher than 0.9 mM (Reis et al., 1990; Johnson and Hallberg, 2008). In addition, *Microbacter* detected in Bio II can also produce acetate and has been previously found in anaerobic acidic sediments (Sánchez-Andrea et al., 2014b).

The removal of sulfate in both bioreactors was variable during the experiment. In the first 100 days of the operation in Bio I, sulfate concentration was below 20 mM, and in the last 110 days of the experiment, the concentration ranged from 4.7 to 18 mM (Figure 1A), unlike Bio II, which maintained the sulfate concentration above 20 mM, except in the last 100 days when the concentration decreased, staying in a range of 5.6–18.7 mM (Figure 1B). These variations in sulfate removal might be explained due to that the consortium in Bio I stabilizes its sulfidogenic population in a shorter period than in Bio II. The range of sulfate concentration observed in the last 110 days of Bio I was similar to that described by Santos and Johnson (2017), in which at a pH of 4 (35°C), sulfate concentration was 6.5–20 mM. Since both reactors had a priming phase (up to day 75 for Bio II), the mean and standard deviation for sulfate measured in the effluent from day 75 for Bio I was 17 and 4 mM (51 sampling points for 267 days of operation). Even considering the variability, the data obtained reflects that the maximum amount of sulfate that can be removed based in carbon source added. Note that, 5 mM of glycerol can remove up to ∼9 mM of sulfate using the stoichiometry glycerol:sulfate (4:7) that described biosulfidogenesis. In addition, the yeast extract added can remove ∼3 mM. Therefore, the amount of sulfate removed is 12 mM (fed with 30 mM). Based in this calculation, the amount of sulfate in the reactor expected would be 18 mM which is close to the mean (17 mM). Similar results were obtained for Bio II.

Protons and carbon sources limit the reduction of sulfate in acidophilic sulfidogenic bioreactors. Nancucheo and Johnson (2014) showed removal of 98% of sulfate (fed with 20 mM) when the pH of the feed liquor was decreased from 3 to 1.6 and glycerol was provided as an electron donor (up to 30 mM). Eq. 1 described by Kimura et al. (2006) shows that the reduction of sulfate to H_2_S is proton-consuming, therefore the partial removal of sulfate achieved in this study suggests a limitation of protons and glycerol supply.

$$4C_{3}H_{8}O_{3}+3SO_{4}^{2-}+6H^{+}\to 4CH_{3}COOH+4CO_{2}+3H_{2}S+8H_{2}O$$ (1)

Organisms from the phyla Firmicutes, Bacteroidetes, Proteobacteria, and Actinobacteria are generally found in bioreactors with the same configuration and objective as those studied here (Nancucheo and Johnson, 2012; Sánchez-Andrea et al., 2014a; Sato et al., 2019). Bio I presented as a dominant microorganism in most samples an SRB, *Desulfosporosinus* which is commonly found in anaerobic acidophilic bioreactors used in AMD treatment (Sánchez-Andrea et al., 2012; Santos and Johnson, 2017; Sato et al., 2019; Hernández et al., 2022). *Desulfosporosinus* is able to form spores under stress conditions, highly relevant in extreme environments found in mine sites (Sato et al., 2019). Moreover, the genus *Desulfosporosinus* is the only validated genus of mesophilic SRB with two described species able to grow in acidic environments, *D. acidiphilus* and *D. acididurans* (Alazard et al., 2010; Sánchez-Andrea et al., 2015).

Another dominant microorganism in Bio I was *Clostridium*, an anaerobic fermenting microorganism. Some of its representatives use glycerol in fermentative processes, such as *Clostridium pasteurianum* (Biebl, 2001), and some of them produce acetic acid, like *Clostridium beijerinckii* (Chen and Blaschek, 1999). *Clostridium* spp. has also been found in bioreactors for AMD treatment (Sánchez-Andrea et al., 2012) and recently Santos and Johnson (2022) reported the presence of this genus in a sulfidogenic reactor maintained at moderately low pH. Although *Clostridium* is not the dominant microorganism in most samples (see Figure 2A)*,* the competition by carbon source (glycerol) and acetic acid production could be problematic to SRBs in the bioreactor. The positive correlation between acetate and *Clostridium* in PCA and Pearson’s correlation graphs (Figure 3 and Supplementary Figure S4A) showed that this bacterium might ferment glycerol in the bioreactor and generates acetic acid.

In Bio II, besides *Desulfosporosinus*, the other dominant microorganism was from the genus *Microbacter*, a microorganism found in acid rock drainage sediments (Sánchez-Andrea et al., 2014b). The observed diversity changes in both bioreactors and changes in predicted microbial function could explain how Bio I showed high acetate concentration. Interestingly, *Microbacter* had not been previously found in Bio II (Santos and Johnson, 2022), though the specie *Microbacter margulisiae* was described as strictly anaerobic and able to grow between pH 3-7 using a variety of sugars, producing acetate, lactate and propionate as major products of fermentation.

Bio II presents a slightly more diverse consortium than Bio I (Supplementary Figure S3) and a more stable predicted microbial function condition, which could help in AMD’s treatment. This bioreactor showed more stability with microorganisms that use sulfur respiration (Figure 2), presenting a slighter variation between 38%—62%, than Bio I, which varied in 10%—77% (in T2—T6). However, at the end of the experiment, Bio I showed more stability and percentage of SRBs (>83%) in T5—T6 (Figure 2) than Bio II.

## Conclusion

This work has demonstrated how two different acidophilic sulfidogenic consortia obtained from different global locations can be operated under continuous flow mode to treat a synthetic acidic mine water. Both bioreactors showed the presence of the genus *Desulfosporosinus*, a known acidophilic SRB, able to partially oxidize glycerol to acetate, which is not a desirable by-product, though during the operation both sulfidogenic systems showed the presence of other microorganisms with the ability to produce acetate and highlight the role of acetate degraders in acidophilic sulfate-reducing bioreactor.

## Data Availability

The genomic data presented in the study were deposited in the NCBI, SRA run accessions SRR21279213 (BIO I) and SRR21279225 (Bio II) in BioProjects PRJNA872571 (BIO I) and PRJNA873019 (Bio II).
