# Supplementary material for: Comparison of two acidophilic sulfidogenic consortia for the treatment of acidic mine water
Source: Front Bioeng Biotechnol. 2022 Nov 29;10:1048412. doi: 10.3389/fbioe.2022.1048412 (PMC9746616; doi:10.3389/fbioe.2022.1048412)
Supplement: Supplementary file 1 [file DataSheet1.docx]

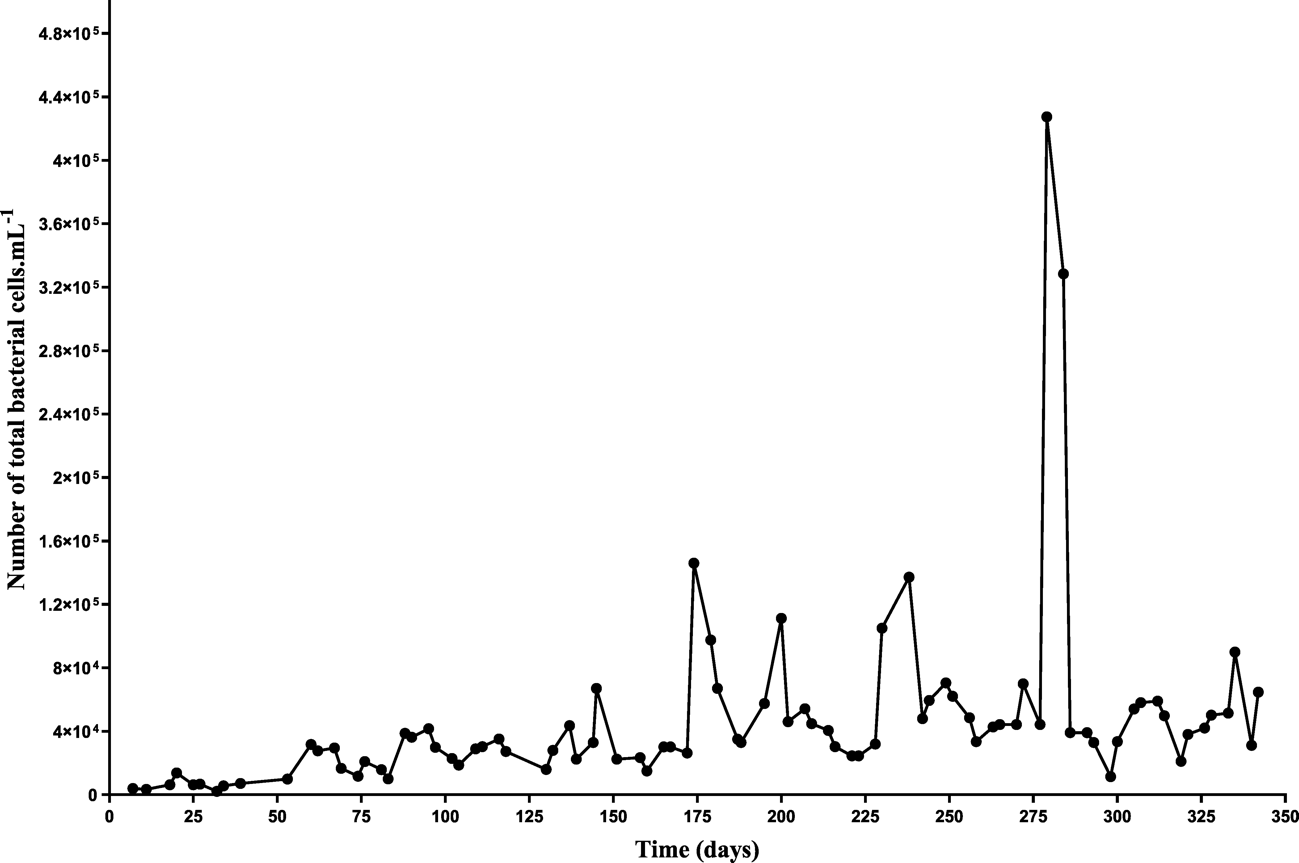


Figure S1. Number of total bacterial cells per mL during the experiment in Bioreactor I (Bio I).


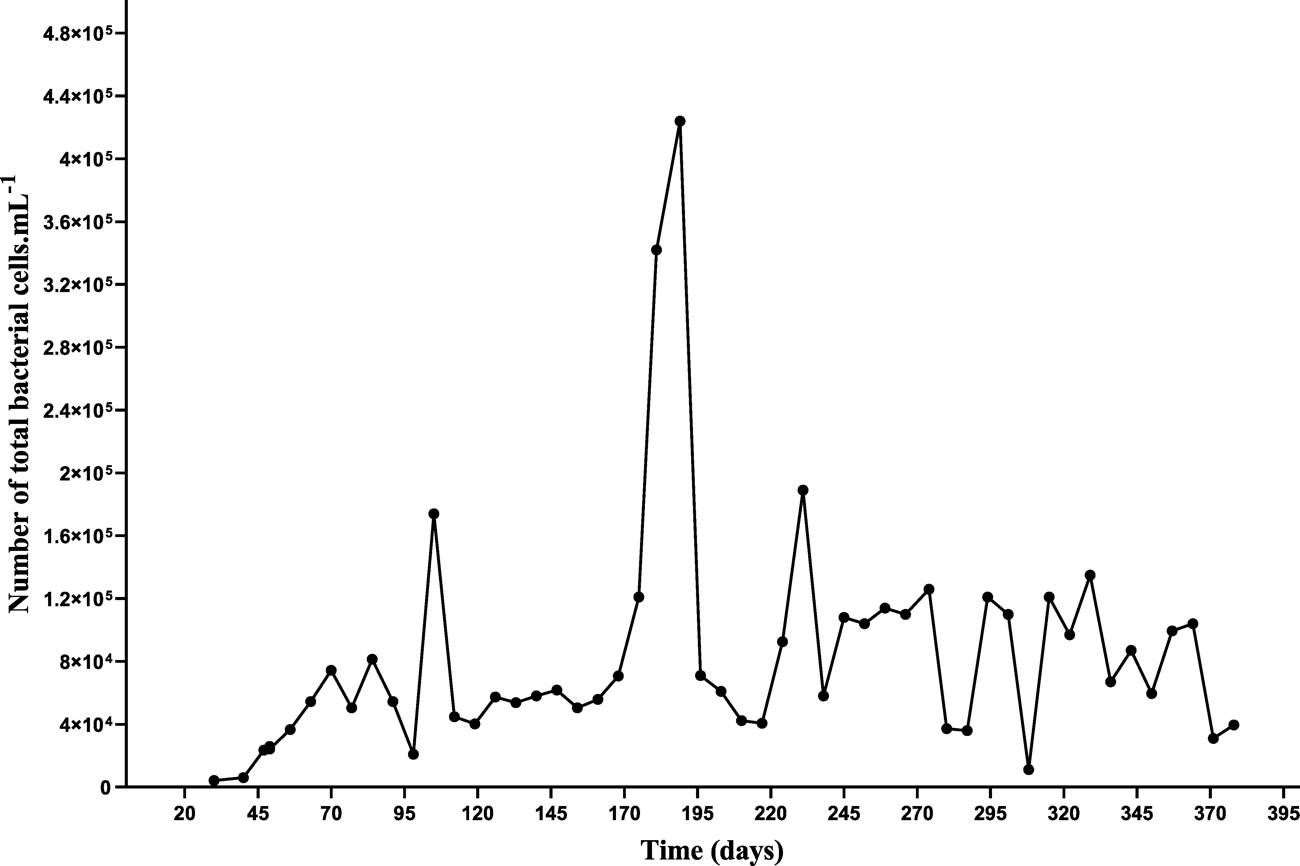


Figure S2. Number of total bacterial cells per mL during the experiment in Bioreactor II (Bio II).


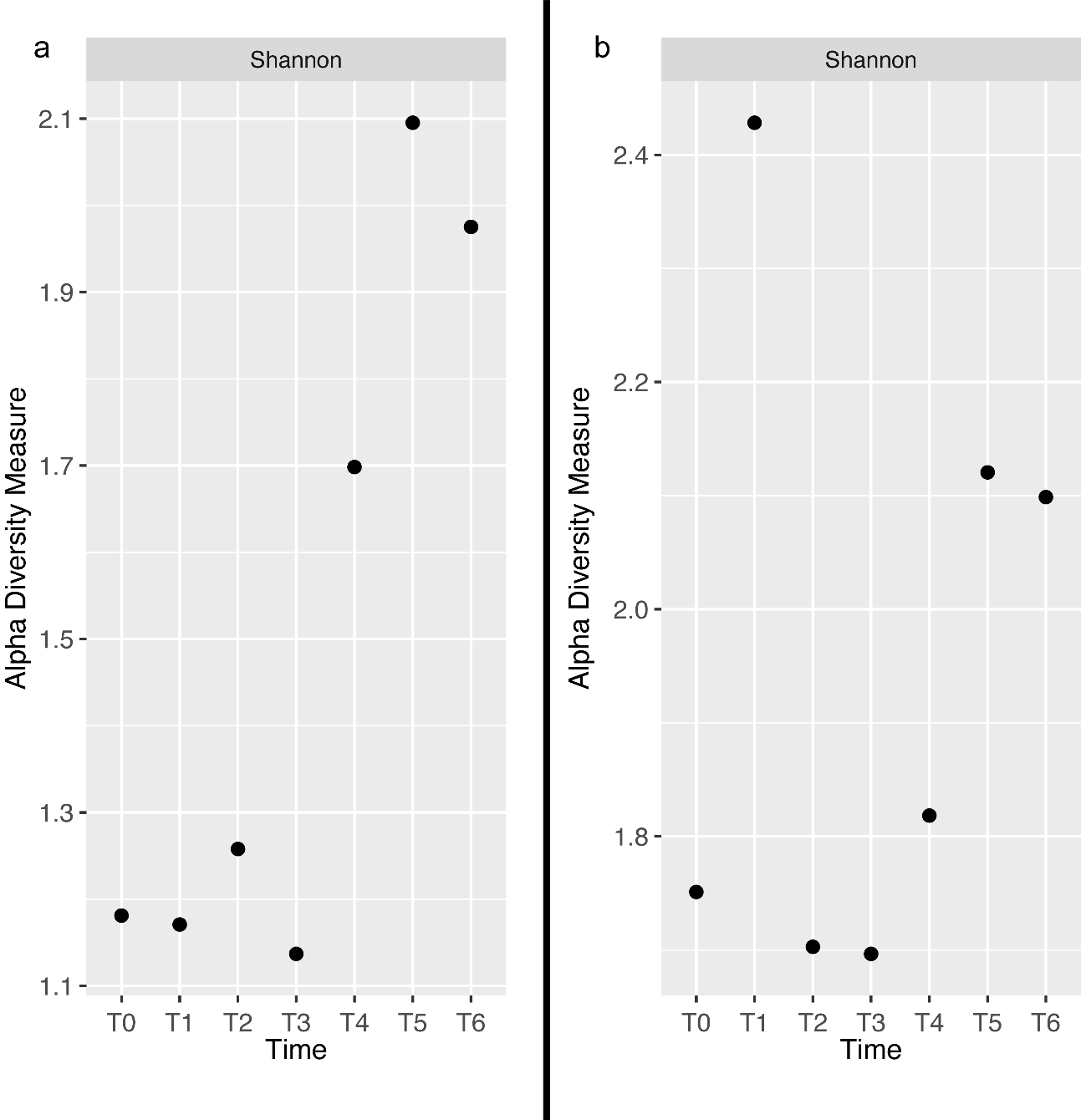


Figure S3. Alpha diversity analysis (Shannon index) of microbial community along time in Bio I (a) and Bio II (b).


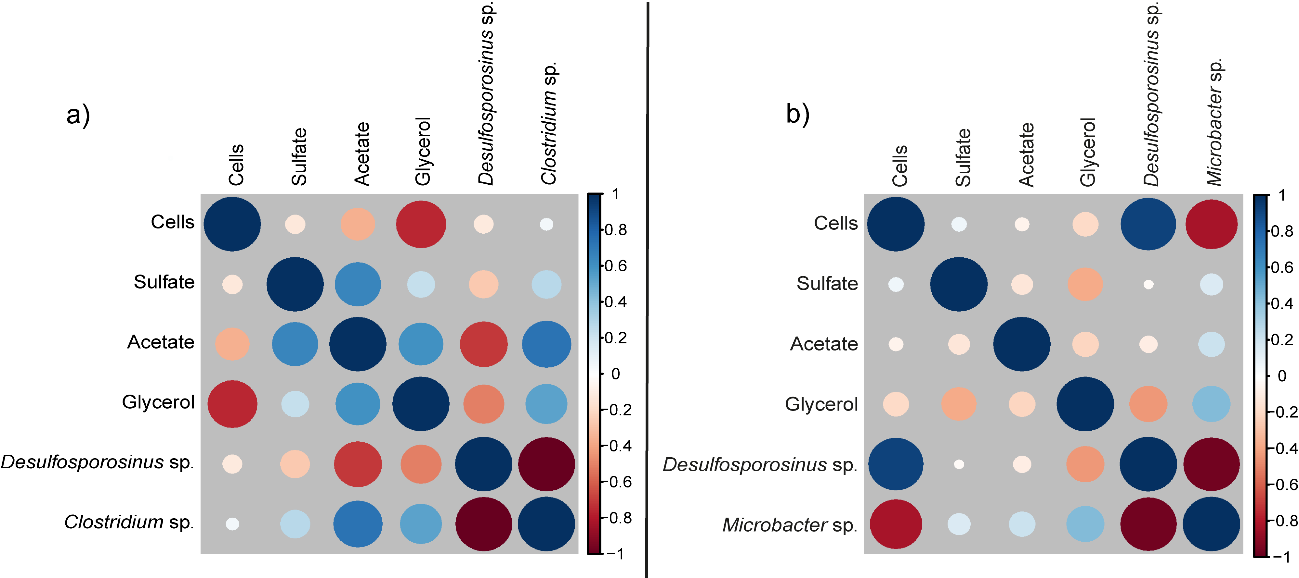


Figure S4: Pearson’s correlation matrix of the total bacterial community based on the relative abundance of bacterial population through 16S rRNA gene sequences and physical-chemical analysis data from Bio I (a) and Bio II (b). Positive and negative correlations are displayed in blue and red circles and color intensity is proportional to the correlation coefficients.
